# Dietary 25-Hydroxyvitamin D_3_ Supplementation Alleviates Porcine Epidemic Diarrhea Virus Infection by Improving Intestinal Structure and Immune Response in Weaned Pigs

**DOI:** 10.3390/ani9090627

**Published:** 2019-08-29

**Authors:** Jiwen Yang, Gang Tian, Daiwen Chen, Ping Zheng, Jie Yu, Xiangbing Mao, Jun He, Yuheng Luo, Junqiu Luo, Zhiqing Huang, Aimin Wu, Bing Yu

**Affiliations:** Institute of Animal Nutrition, Sichuan Agricultural University, NO. 46 Xinkang Road, Yucheng District, Yaan, Sichuan 625014, China

**Keywords:** piglets, vitamin D, antivirus, diarrhea, intestinal morphology

## Abstract

**Simple Summary:**

Porcine epidemic diarrhea is one of the major problems in current swine husbandry worldwide, and effective measures for prevention and treatment are scarce. We found that high dose 25-hydroxyvitamin D_3_ supplementation could ease intestinal injury and inhibit intestinal immune response induced by porcine epidemic diarrhea virus (PEDV), suggesting that feeding a high dose of 25-hydroxyvitamin D_3_ could be used as an approach against PEDV infection.

**Abstract:**

We conducted this experiment to determine if feeding 25-hydroxyvitamin D_3_ (25(OH)D_3_) to weaned pigs would alleviate porcine epidemic diarrhea virus (PEDV) infection and immune response. Forty-two weaned pigs were allotted to 1 of 6 dietary 25(OH)D_3_ treatments (5.5, 5.5, 43.0, 80.5, 118.0, 155.5 μg 25(OH)D_3_/kg diet) for 26 days. On day 22 of the trial, all the treatments were orally administrated with PEDV except for one of the 5.5 μg 25(OH)D_3_/kg treatments, which was challenged with the same volume of sterile saline and served as control. Another 5.5 μg 25(OH)D_3_/kg group for PEDV challenge was named CON-PEDV. Average daily gain (*p* < 0.05) was reduced by PEDV infection. PEDV administration also induced severe diarrhea (*p* < 0.05), reduction of villous height and the ratio of villous height to crypt depth, and increase of crypt depth and serum diamine oxidase activity (*p* < 0.05). Serum IgM and complement component 4 levels were increased by PEDV challenge. However, 155.5 μg 25(OH)D_3_/kg supplementation alleviated intestinal damage (*p* < 0.05) compared with CON-PEDV. Furthermore, 155.5 μg 25(OH)D_3_/kg supplementation downregulated the mRNA abundance of inflammatory cytokines and interferon signal pathway-related genes (*p* < 0.05) compared with CON-PEDV. These results suggested that dietary supplementation of 155.5 μg 25(OH)D_3_/kg could alleviate intestinal damage and protect against PEDV-induced inflammatory status.

## 1. Introduction

Porcine epidemic diarrhea virus (PEDV) infection causes severe damage to the intestinal function and barrier integrity of pigs [1], leading to diarrhea, vomiting, dehydration, and high mortality in piglets [2]. Recently, it was shown that the villus height and transepithelial resistance were decreased in PEDV-infected pigs [3]. PEDV also induced intestinal mucosa and systemic proinflammatory cytokine responses in pigs [4,5]. Currently, vaccination is the main means for preventing PEDV infection. However, vaccines are poorly effective because of genetic variants of the viruses [6,7].

Vitamin D not only enhances calcium and phosphate absorption but also regulates immune function [8]. Previous studies have shown that vitamin D can inhibit rotavirus replication and alleviate infection symptoms in piglets [9,10]. PEDV infection has shown similar symptoms to rotavirus and, therefore, we speculated that 25(OH)D_3_ might ease the infection of PEDV in pigs. However, studies on the feasibility of vitamin D_3_ as anti-PEDV infection agent in piglets are scarce.

It is generally believed that the bioavailability of 25(OH)D_3_ is higher than vitamin D_3_ [11,12,13]. In this study, 25(OH)D_3_ was used to investigate whether it could alleviate PEVD-infected diarrhea and intestinal injury.

## 2. Materials and Methods

The experimental protocol involved in the present study was approved by the Animal Care Advisory Committee of Sichuan Agricultural University (Animal Ethics Committee approval number is CD-SYXK-2017-015).

### 2.1. Experimental Design

Forty-two crossbred healthy weaned pigs (Duroc × Landrace × Yorkshire, 24 days old) with an initially body weight (BW) of 6.61 ± 0.41 kg were used in the 26 days trial. On the first day of the trial, all pigs were allotted on the basis of BW to six groups and each fed diets supplemented with either 5.5, 5.5, 43.0, 80.5, 118.0, or 155.5 μg 25(OH)D_3_/kg. Each treatment consisted of four gilts and three barrows. At day 22 of this study, all the treatments were orally administrated with 35 mL of PEDV (5.6 × 10^3^ TCID_50_/mL) except for one of the 5.5 μg 25(OH)D_3_/kg (220 IU vitamin D/kg equivalent) treatment, which was served as control (CON) and administrated with the same volume of sterile saline. Another 5.5 μg 25(OH)D_3_/kg treatment for PEDV challenge was named by CON-PEDV. In order to prevent infection, the CON groups were housed in the next room of challenge groups. The settings for the two rooms were the same. Pigs in the CON group were negative for PEDV throughout the trial period. As shown in Table 1, the basal diet was formulated to meet or exceed nutrient requirement for weaned piglets [14], except for vitamin D_3_, which was not prepared in the vitamin premix. Each treatment was formed by supplementing with indicated 25(OH)D_3_ levels in the basal diet. All pigs had ad libitum access to water and experimental diets throughout the trial. 25(OH)D_3_ (Hy-D) was kindly provided by DSM Nutritional Products Ltd. Shanghai, China. The PEDV was kindly presented by professor Zhiwen Xu, College of Veterinary Medicine, Sichuan Agricultural University. Average daily gain (ADG) and average daily feed intake (ADFI) were determined via weighing to determine body weight and recording of feed intake.

After the PEDV challenge, the fecal consistency and diarrhea incidence were assessed every day according to Hu et al. [15]: 1 = hard feces, 2 = firm well formed, 3 = soft and partially formed feces, 4 = loose, semi-liquid feces, and 5 = watery feces.

Diarrhea rate (%) = (A/5d) × 100, in which A = total days per pig with diarrhea after PEDV challenge.

Mean cumulative score = ∑A/B, in which A = the sum of daily scores, and B = pigs per treatment.

### 2.2. Sample Collection

All pigs were bled via anterior vein on 27 day. The blood was used for extracting serum via centrifugation at 3000 *g* for 15 min, and the serum samples were stored at −20 °C until analysis. All pigs were then euthanized by intramuscular injection of Shumianning (comprised of ketamine, xylazine, and midazolam, Nanjing Agricultural University, 0.08 mL/kg body weight). About 2 cm jejunal tissue sample was stored in 4% paraformaldehyde solution for histological analysis. Mucosal samples from the middle jejunum were scraped and rapidly frozen in liquid nitrogen, and then stored at −80 °C for further analysis.

### 2.3. Immunological Parameters

The concentration of IgG, IgM, and complement component 3 (C3) and C4 (Sichuan Maker Biotechnology Co. Ltd. Chengdu, China) in serum were detected by automatic biochemical analyzer (Model 3100; Hitachi, Tokyo, Japan). Immunology multiple control were performed before sample determination to ensure the outcomes were correct.

### 2.4. Intestinal Morphology and Integrity

After being embedded in paraffin, the jejunal samples were stained with hematoxylin and eosin for intestinal morphology measurement. A minimum of 20 well-orientated villi and crypts from each intestinal sample of pigs were measured using Image-Pro Plus 6.0 software. As a measurement of intestinal permeability, serum diamine oxidase activity (DAO) was detected using commercial assay kits (Nanjing Jiancheng Institute of Bioengineering, Jiangsu, China) following the protocols of the manufacturer.

### 2.5. Gene Expression

Total RNA was extracted from the mucosa of jejunum tissue using TRIzol reagent (Invitrogen, Shanghai, China). Reverse transcription was performed with RNA using a PrimeScript RT reagent kit (TaKaRa, Dalian, China). The mRNA expression of genes of interest were quantified using an ABI 7900HT detection system (Applied Biosystems, Foster, CA, USA) and the SYBR Premix Ex Taq II with ROX reagents (TaKaRa, Dalian, China). The primer sequences used for RT-PCR are listed in Table 2. All primer pairs were designed to have melting temperatures of approximately 60 °C. Cycling conditions were as follows: 95 °C for 30 s, followed by 40 cycles of 95 °C for 5 s and 60 °C for 30 s. The relative mRNA expression of each gene was calculated according to a previous publication [16]. Expression levels were normalized to *β-actin*.

### 2.6. Protein Expression

Protein sample processing was performed according to Zhang et al. [17]. Briefly, jejunal mucosa protein was extracted with lysis buffer (Beyotime, Shanghai, China). After centrifugation for 20 min at 12,000 rpm, the supernatants were harvested for bicinchoninic acid assay to detect protein concentration. Then, the samples were separated by sodium dodecyl sulfate-polyacrylamide gel electrophoresis and transferred to polyvinylidene fluoride membranes, and incubated with the corresponding antibodies: anti-sucrase-isomaltase (SI), anti-sIgA, anti-occludin (Abcam, Shanghai, China), and anti-β-actin (Santa Cruz, Shanghai, China). Following washing, the samples were incubated with secondary antibodies, and then proteins were incubated with Electro-Chemi-Luminescence reagent for chemiluminescence. The protein expression was analyzed by Image Lab and normalized to β-actin.

### 2.7. Statistical Analyses

In this study, each pig was used as the experimental unit. The differences between CON and CON-PEDV group were assayed by Student’s *t*-test. On the condition of PEDV challenge, the data were performed using the PROC MIXED SAS 9.4 procedure according to the following model:

Y = μ + α + β + ε,

where Y = dependent variable; μ = mean; α = effect of treatment; β = block effect of BW, and ε = error. Orthogonal comparisons were also applied for linear and quadratic responses of increasing dietary 25(OH)D3 levels (5.5, 43.0, 80.5, 118.0, 155.5 μg 25(OH)D3/kg). Data of the innate immune gene expression were analyzed by Tukey’s test for post hoc comparisons. The significance was declared at *p* < 0.05 and trends at *p* < 0.10.

## 3. Results

### 3.1. Performance and Diarrhea Parameter

As shown in Table 3, PEDV challenge (CON-PEDV) decreased ADG (*p* < 0.05) and ADFI (*p* = 0.08) compared with CON. However, the performance was not influenced among the different 25(OH)D_3_ supplementation groups. PEDV infection induced severe diarrhea in piglets (Table 3, *p* < 0.05). However, the dietary supplementation of 25(OH)D_3_ decrease diarrhea scores (*p* < 0.05) and diarrhea rate (Table 3, *p* < 0.1).

### 3.2. Immunological Responses

PEDV infection increased serum IgM and C4 concentrations compared with CON (*p* < 0.05, Table 4). However, different levels of 25(OH)D_3_ supplementation had no effect on serum IgM, IgG, and C4 levels on the condition of PEDV challenge. In addition, dietary supplementation of 155.5 μg 25(OH)D_3_/kg decreased serum C3 levels compared with the 118.0 μg 25(OH)D_3_/kg group. 

### 3.3. Intestinal Morphology and Permeability

Compared with CON, PEDV challenge decreased villous height and the ratio of villous height to crypt depth (VCR), and increased the crypt depth of jejunum in the CON-PEDV group (Table 5, *p* < 0.05). However, 118.0 and 155.5 μg 25(OH)D_3_/kg treatments significantly increased villous height and VCR and decreased crypt depth compared with the CON-PEDV group (Table 5, *p* < 0.05). Furthermore, dietary supplementation of 25(OH)D_3_ increased villous height and VCR and decreased crypt depth in a linear way (*p* < 0.05).

**Figure 1:**
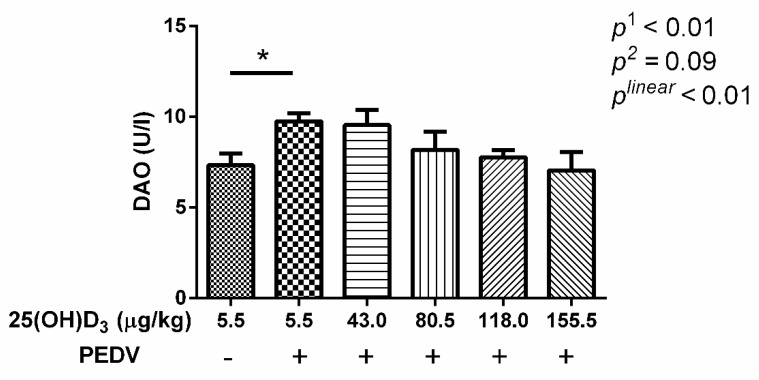
Serum diamine oxidase activity (DAO) activity of weaned piglets fed 25(OH)D3 as indicated with PEDV challenge. The *p*_1_ means *t*-test between CON and CON-PEDV, and *p*_2_ value means multiple comparisons among the PEDV challenge groups.

In addition, serum DAO activity of pigs in the CON-PEDV group was significantly increased compared with CON (Figure 1, *p* < 0.05). However, the dietary supplementation of 25(OH)D_3_ tended to decrease serum DAO activity (*p* < 0.1).

### 3.4. Intestinal Barrier Related Genes and Protein Expression

**Figure 2:**
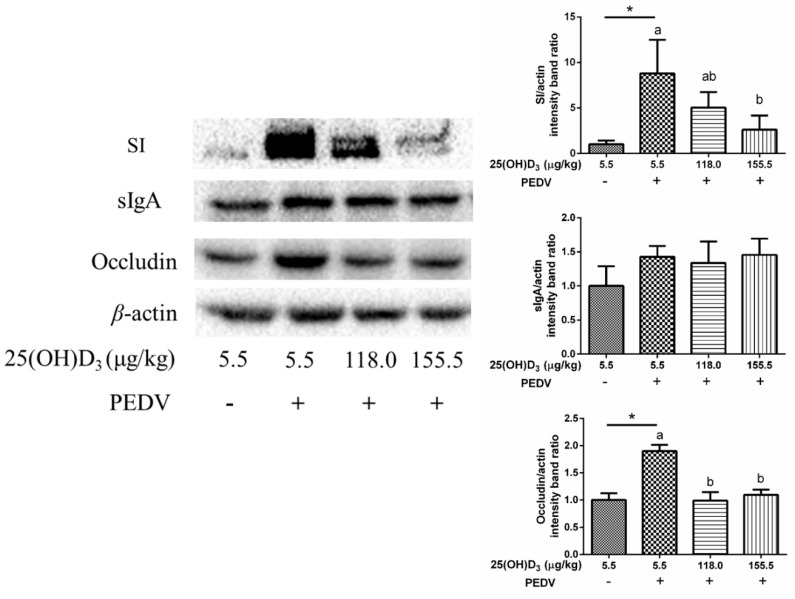
Intestinal barrier related protein expression in the jejunal mucosa of weaned piglets fed indicated 25(OH)D_3_ dietary with PEDV challenge.

Compared with CON, the gene expression of intestinal barrier-related genes was not influenced by PEDV (Table 6). Upon PEDV challenge, the dietary supplementation of 25(OH)D_3_ increased jejunal *claudin-2* gene expression significantly and this treatment effect was linear (*p* < 0.05). 25(OH)D_3_ also tended to increase *MUC2* and mRNA expression (*p* < 0.1). Interestingly, PEDV increased sucrase-isomaltase (SI) and occludin protein levels, but high doses 25(OH)D_3_ (155.5 μg 25(OH)D_3_/kg) inhibited these increase (Figure 2).

### 3.5. Innate Immune Gene Expression in Jejunum

PEDV induced a significant increase in the mRNA expression of *RIGI*, *TLR2*, *MyD88*, *IL6*, *IL8*, *IFNλ1*, and *MxA* compared to CON (Table 7, *p* < 0.05). However, a high dose of dietary 25(OH)D_3_ supplementation decreased *TLR2*, *TLR9*, *MyD88*, *IL6*, *IL8*, *IFNλ1*, *STAT1*, *MxA*, *IFNAR1*, and *TRIF* mRNA expression compared with CON-PEDV (Table 7, *p* < 0.05).

## 4. Discussion

Early studies suggested that vitamin D exerts broad-spectrum antiviral effects, including inhibiting the replication of dengue virus [18], hepatitis C virus [19], rotavirus [9], and others. Due to the similarity in symptoms after rotavirus and PEDV infection in pigs, we carried out this study to investigate whether 25(OH)D_3_ could alleviate PEDV infection.

We have previously demonstrated that increasing dietary 25(OH)D_3_ levels linearly increased serum 25(OH)D_3_ concentrations, but no treatment effects were observed in the growth performance of weaned pigs [20]. In the current study, PEDV challenge decreased ADG and ADFI, and resulted in severe diarrhea of the piglets. Previous studies have reported that PEDV infection reduced growth performance and resulted in severe diarrhea [21,22,23]. We have analyzed the PEDV-N gene in jejunum mucosa by PCR, and the results revealed that PEDV was prevalent in the PEDV-inoculated pigs, whereas CON treatments were negative for PEDV (Supplementary Materials). This suggested that the PEDV infection model was successfully established. In the current study, dietary 25(OH)D_3_ supplementation decreased diarrhea scores, and 155.5 μg 25(OH)D_3_/kg treatment showed the minimum diarrhea scores and diarrhea rate, which indicated that supplementation with a high dose of 25(OH)D_3_ might alleviate the symptoms of PEDV infection.

The complement system is a part of the innate immune system, and not only participates in inflammation but also enhances the adaptive immune response [24]. In this study, serum C4 level was increased with PEDV challenge as compared with CON. We also found that serum IgM levels were increased by PEDV challenge. Immunoglobulins are the major secretory products of humoral immunity [25]. These results indicated that the PEDV infection activated the innate and humoral immune response. However, dietary 25(OH)D_3_ supplementation had no effect on serum C4 and IgM levels under the conditions of PEDV challenge. We inferred that 25(OH)D_3_ might exert an antiviral effect on intestinal mucosa, which is the main target of PEDV replication [26].

PEDV infection always leads to morphological changes of the small intestine with a reduction in villus height and damage to intestinal integrity [3,27]. In this study, we also found that villus height and VCR were decreased, and crypt depth was increased with PEDV challenge. In addition, serum DAO activity was also increased by PEDV challenge. Serum DAO activity is a marker of mucosal integrity [28]. These results indicated that PEDV induced morphological changes of jejunum and an increase in intestinal permeability. The 155.5 μg 25(OH)D_3_/kg 25(OH)D_3_ treatment was shown to be optimal in alleviating intestinal injury induced by PEDV. Therefore, this may be the reason why the 155.5 μg 25(OH)D_3_/kg treatment showed the minimum diarrhea scores and diarrhea rate among the treatments. Moreover, we found that *claudin2* gene expression showed a linear response with increasing dietary 25(OH)D_3_ concentration, and 25(OH)D_3_ tended to increase *MUC2* expression under the conditions of PEDV challenge. It suggested that high dose 25(OH)D_3_ supplementation could improve tight junction protein expression to maintain intestinal barrier integrity. Interestingly, levels of SI, an intestinal absorptive cell marker, were significantly increased by PEDV challenge. A previous study has shown that PEDV infection decreased goblet cells in intestinal villous [29]. Hence, we infer that PEDV infection could cause disorder differentiation of intestinal cells, which promotes the differentiation of intestinal stem cells to intestinal absorption cells, while decreasing differentiation to intestinal secretion cells. However, high dose 25(OH)D_3_ supplementation significantly reduced the SI expression induced by PEDV, which might be beneficial for maintaining normal intestinal function. We also found occludin expression was increased with PEDV challenge compared with CON, but 118.0 and 155.5 μg 25(OH)D_3_/kg groups inhibited this increase. Luo et al. [30] demonstrated that overexpression of occludin in target cells makes them more susceptible to PEDV infection, which indicated that occludin plays an essential role in PEDV infection. In the present study, we speculated that 155.5 μg 25(OH)D_3_/kg supplementation might ease PEDV infection through decreasing occludin expression.

Innate immune response plays an important role in defense against viral infections in mammalian cells. During viral infection, the virus is recognized by pattern-recognition receptors (PRRs) including toll-like receptors (TLRs) and retinoic acid-inducible gene I (RIGI) or melanoma differentiation gene 5 (MDA5), then INFs and proinflammatory cytokines are produced for initiation of the inherent antiviral immune response [31,32]. Different types of IFNs bind to different receptors. Type I IFNs (IFN-α and IFN-β) signal through IFNAR1 and IFNAR2 to activate the JAK-STAT signaling pathway. And type III IFNs (IFN-λs) signal through IFNLR1 and IL10R2 to activate JAK-STAT signaling pathway to induce the expression of hundreds of interferon stimulating genes [31]. Unlike type I interferon receptors, which are seemingly ubiquitous, type III IFN receptors are confined to the mucosal epithelium [33]. Thus, IFN-λs mainly play an antiviral role in mucosal epithelial cells. In the current study, PEDV increased the PRR, inflammatory cytokine, and *IFNλ* expression in the jejunum mucosa. This indicates that the IFNs signaling pathway was activated by PEDV in the intestine of piglets. Since the high dose of 25(OH)D_3_ supplementation showed a better protective effect than the low dose groups, we investigated whether high dose supplementation could alleviate PEDV infection by regulating immunity. We found that a high dose of 25(OH)D_3_ inhibited the PRR, *IFNλ*, *STAT1*, and *MxA* expression. It was suggested that dietary 25(OH)D_3_ supplementation inhibited the activation of intestinal immunity induced by PEDV. Previous studies have shown that vitamin D attenuated rotavirus infection and reduced the viability of *Mycobacterium tuberculosis* through regulating autophagy and cathelicidin [9,34]. Therefore, we speculated that the suppression of the IFN signaling pathway from high dose supplementation of 25(OH)D_3_ might be due to decreased PEDV replication. In addition, we also found that high doses of 25(OH)D_3_ inhibited jejunal mucosa *IL6* and *IL8* mRNA expression compared with CON-PEDV. This suggested that high dose 25(OH)D_3_ supplementation might inhibit intestinal inflammation induced by PEDV. Reducing the expression of intestinal inflammatory cytokines is also beneficial in maintaining normal intestinal function.

## 5. Conclusions

In summary, the results of the current study indicate that dietary supplementation of 155.5 μg/kg 25(OH)D_3_ alleviated the severity of diarrhea of piglets infected with PEDV by improving the intestinal structure and immune response, and maintaining regular intestinal function.

## Figures and Tables

**Table 1:** Ingredients and nutrient contents of the basal diet.

Items	Content
Ingredients (%)	
Corn	35.28
Corn, extruded	26.00
Soybean meal, dehulled	9.50
Soybean meal, extruded	8.00
Soybean protein concentrate	5.50
Sucrose	2.00
Soybean oil	1.50
Fish meal	3.50
Dried whey	5.50
L-Lysine HCl	0.53
DL-Methionine	0.10
L-Threonine	0.13
L-Tryptophan	0.03
Limestone	0.90
Dicalcium phosphate	0.45
Choline chloride	0.15
NaCl	0.20
Benzoic acid	0.50
vitamin premix ^1^	0.03
Mineral premix ^2^	0.20
Total	100.00
Nutrient content (%)	
Digestible energy (kJ/kg)	14.64
Crude protein	18.6
Calcium	0.81
STTD ^3^ phosphorus	0.37
SID ^4^ Lys	1.40
SID ^4^ Met	0.40
SID ^4^ Thr	0.80
SID ^4^ Trp	0.22

^1^ Provided per kilogram of complete diet: 10,050 IU vitamin A; 40 IU vitamin E; 4 mg vitamin K3; 3 mg thiamine; 9 mg riboflavin; 6 mg VB6; 0.04 mg VB12; 0.3 mg biotin; 35 mg nicotinic acid; 15 mg pantothenic acid; 1.5 mg folic acid. ^2^ Provided per kilogram of complete diet: 100 mg Fe; 10 mg Cu; 20 mg Mn; 0.3 mg I; 100 mg Zn; Se 0.3 mg. ^3^ Standardized total tract digestible. ^4^ Standardized ileal digestible.

**Table 2:** Primer sequence of the target and reference genes.

Genes	Primer Sequence (5′–3′)	Product	GenBank Accession
*MUC2*	Forward: GGTCATGCTGGAGCTGGACAGT	181	XM_021082584.1
	Reverse: TGCCTCCTCGGGGTCGTCAC		
*Claudin2*	Forward: GCATCATTTCCTCCCTGTT	156	NM_001161638.1
	Reverse: TCTTGGCTTTGGGTGGTT		
*ZO1*	Forward: CGTGTCAACGCCACTATCA	90	XM_021098896.1
	Reverse: TTGTCTTCCAAAGCCCCT		
*Occludin*	Forward: AACTTCCACTGATGTCCCCCGT	116	NM_001163647.2
	Reverse: CCTAGACTTTCCTGCTCTGCCC		
*RIGI*	Forward: AGAGCAGCGGCGGAATC	82	NM_213804.2
	Reverse: GGCCATGTAGCTCAGGATGAA		
*MDA5*	Forward: TCCGGGAAACAGGCAACTC	75	NM_001100194.1
	Reverse: CAAAGGATGGAGAGGGCAAGT		
*TLR2*	Forward: TCACTTGTCTAACTTATCATCCTCTTG	162	XM_005653576.3
	Reverse: TCAGCGAAGGTGTCATTATTGC		
*TLR9*	Forward: CACGACAGCCGAATAGCAC	121	NM_213958.1
	Reverse: GGGAACAGGGAGCAGAGC		
*MyD88*	Forward: GTGCCGTCGGATGGTAGTG	65	NM001099923
	Reverse: TCTGGAAGTCACATTCCTTGCTT		
*IFNα*	Forward: ACCTGGAAGCCTGTGTCATG	164	NM_214393.1
	Reverse: CATGACTTCTGCCCTGACGA		
*IFNλ*	Forward: TGCATCACATCCACGTCGAA	131	NM_001142837.1
	Reverse: GCAGCCTTGGGACTCTTTCT		
*MxA*	Forward: GCATCACCAGGGTAGCTGTA	195	NM_214061.2
	Reverse: AGATCCCGATGGTCCTGTCT		
*IL6*	Forward: TTCACCTCTCCGGACAAAAC	122	NM_001252429.1
	Reverse: TCTGCCAGTACCTCCTTGCT		
*IL8*	Forward: AGTTTTCCTGCTTTCTGCAGCT	72	NM_213867.1
	Reverse: TGGCATCGAAGTTCTGCACT		
*STAT1*	Forward: GCAGGTTCATCAGCTCTACGA	124	NM_213769.1
	Reverse: AAAACGGATGGTGGCAAACG		
*IFNAR1*	Forward: TCTTCATCCGTGTCCAAGCA	105	NM_213772.1
	Reverse: TGATGACGGGAGGAAACAGG		
*IFNLR1*	Forward: TCCGAGGACTTGAGTTCCCT	126	XM_021095536.1
	Reverse: GGTCAGTGTCCGCAGAGAAA		
*TRIF*	Forward: ACTCGGCCTTCACCATCCT	87	NM_001315738.2
	Reverse: GGCTGCTCATCAGAGACTGGTT		
*TRAF3*	Forward: ACACCGGCTACTTTGGCTAC	106	XM_021081630.1
	Reverse: TCCCCACGCATGATGACAAA		
*β-Actin*	Forward: GGATGACGATATTGCTGCGC	190	XM_003124280.5
	Reverse: GATGCCTCTCTTGCTCTGGG		

MUC2 = mucin 2; ZO1 = zonula occludens protein-1; RIGI = retinoic acid inducible gene I; MDA5 = melanoma differentiation-associated protein 5; TLR = toll-like receptor; MyD88 = myeloid differentiation factor 88; IFN = interferon; MxA = myxovirus resistance A; IL = interleukin; STAT = signal transducers and activators of transcription; IFNAR1 = interferon α and β receptor subunit 1; IFNLR1 = interferon lambda receptor 1; TRIF = toll-like receptor-associated activator of interferon; TRAF3 = TNF receptor-associated factor 3.

**Table 3:** Growth performance and diarrhea parameter of weaned piglets fed 25(OH)D_3_ as indicated with porcine epidemic diarrhea virus (PEDV) challenge.

	CON (PEDV−)	PEDV Challenge					SEM	*p*-Value		
25(OH)D_3_ (μg/kg)	5.5	5.5	43.0	80.5	118.0	155.5		25(OH)D_3_	Linear	Quadratic
1–26 day										
ADG (g)	301.40	246.62 *	220.27	252.12	282.36	256.41	23.39	0.46	0.29	0.99
ADFI (g)	456.25	401.86 ^#^	374.90	399.64	433.40	376.48	22.95	0.24	0.81	0.49
Feed efficiency	0.66	0.61	0.57	0.63	0.64	0.67	0.02	0.24	0.07	0.33
22–26 day										
Mean cumulative score	10.00	16.21 *^,a^	15.71 ^a,b^	13.93 ^a,b^	14.00 ^a,b^	11.25 ^b^	1.27	0.04	<0.01	0.57
Diarrhea rate (%)	0	60.00 ^*^	54.29	48.57	48.57	16.67	10.25	0.06	<0.01	0.24

SEM, standard error. * Means different from CON (*p* < 0.05); ^#^ Means different from CON (*p* < 0.1); ^a,b^ Means not sharing the same superscript differ at *p* < 0.05.

**Table 4:** Serum immunoglobulins and complement component levels of weaned piglets fed 25(OH)D_3_ as indicated with PEDV challenge.

	CON (PEDV−)	PEDV Challenge					SEM	*p*-Value		
25(OH)D_3_ (μg/kg)	5.5	5.5	43.0	80.5	118.0	155.5		25(OH)D_3_	Linear	Quadratic
IgG (g/L)	3.63	4.34	4.68	4.33	3.84	4.45	0.25	0.26	0.43	0.66
IgM (mg/L)	96.71	204.43 *	156.71	165.83	222.86	216.43	34.93	0.53	0.39	0.32
C3 (mg/L)	64.14	75.86 ^a,b^	76.71 ^a,b^	64.57 ^a,b^	87.83 ^a^	58.14 ^b^	6.02	0.02	0.21	0.26
C4 (mg/L)	4.17	8.17 *	13.57	15.86	11.43	10.14	2.53	0.27	0.83	0.04

SEM, standard error; * Means different from CON (*p* < 0.05); ^a,b^ Means not sharing the same superscript differ at *p* < 0.05.

**Table 5:** Jejunal villous height, crypt depth, and the ratio of villous height/crypt depth (VCR) of weaned piglets fed 25(OH)D_3_ as indicated with PEDV challenge.

	CON (PEDV−)	PEDV Challenge					SEM	*p*-Value		
25(OH)D_3_ (μg/kg)	5.5	5.5	43.0	80.5	118.0	155.5		25(OH)D_3_	Linear	Quadratic
Villus height (μm)	468.40	263.19 *^,b^	290.2 ^a,b^	314.51 ^a,b^	330.56 ^a^	333.36 ^a^	14.27	0.01	<0.01	0.26
Crypt depth (μm)	189.40	247.9 *^,a^	219.46 ^b^	190.03 ^b,c^	179.64 ^c,d^	163.6 ^d^	5.42	<0.01	<0.01	0.11
VCR	2.50	1.06 *^,a^	1.39 ^a,b^	1.65 ^b,c^	1.84 ^c,d^	2.02 ^d^	0.09	<0.01	<0.01	0.25

SEM, standard error; * Means different from CON (*p* < 0.05); ^a,b,c,d^ Means not sharing the same superscript differ at *p* < 0.05.

**Table 6:** Relative gene expression of intestinal barrier-related genes of weaned piglets fed the indicated 25(OH)D_3_ with PEDV challenge.

	CON (PEDV−)	PEDV Challenge					SEM	*p*-Value		
25(OH)D_3_ (μg/kg)	5.5	5.5	43.0	80.5	118.0	155.5		25(OH)D_3_	Linear	Quadratic
*MUC2*	1.00	1.22	0.98	0.92	1.37	1.71	0.17	0.09	0.02	0.02
*Occludin*	1.00	1.10	0.83	1.07	1.23	1.63	0.28	0.35	0.11	0.20
*ZO1*	1.00	1.13	0.76	0.81	0.92	1.34	0.20	0.24	0.38	0.04
*Claudin2*	1.00	1.02	1.02	1.08	1.29	1.33	0.09	0.04	<0.01	0.52

SEM, standard error.

**Table 7:** Relative gene expression of innate immune in the jejunal mucosa of weaned piglets fed 25(OH)D_3_ as indicated with PEDV challenge.

	CON (PEDV−)	PEDV Challenge			*p* ^1^	*p* ^2^
25(OH)D_3_ (μg/kg)	5.5	5.5	118.0	155.5		
*RIG1*	1.00 ± 0.20	3.07 ± 0.68 *	1.94 ± 0.38	1.41 ± 0.30	0.02	0.09
*MDA5*	1.00 ± 0.20	1.27 ± 0.23	0.83 ± 0.09	0.73 ± 0.07	0.38	0.24
*TLR2*	1.00 ± 0.14	3.94 ± 0.72 *^,a^	1.77 ± 0.54 ^b^	1.58 ± 0.37 ^b^	<0.01	0.02
*TLR9*	1.00 ± 0.20	1.40 ± 0.19	1.06 ± 0.12	0.81 ± 0.18	0.17	0.07
*MyD88*	1.00 ± 0.10	1.42 ± 0.12 *	1.13 ± 0.12	1.16 ± 0.12	0.02	0.20
*IFNα*	1.00 ± 0.11	0.94 ± 0.13	1.02 ± 0.18	0.93 ± 0.13	0.72	0.9
*IFNλ*	1.00 ± 0.23	2.06 ± 0.32 *^,a^	1.20 ± 0.25 ^b^	1.10 ± 0.10 ^b^	0.02	0.02
*MxA*	1.00 ± 0.24	2.96 ± 0.61 *^,a^	1.72 ± 0.17 ^a,b^	1.05 ± 0.18 ^b^	0.01	0.02
*IL6*	1.00 ± 0.20	2.49 ± 0.52 *^,a^	0.97 ± 0.28 ^b^	0.71 ± 0.18 ^b^	0.04	0.01
*IL8*	1.00 ± 0.20	2.73 ± 0.54 *^,a^	1.53 ± 0.50 ^a,b^	0.95 ± 0.11 ^b^	0.02	0.03
*STAT1*	1.00 ± 0.17	1.25 ± 0.16 ^a^	0.92 ± 0.11 ^a,b^	0.67 ± 0.13 ^b^	0.31	0.03
*IFNAR1*	1.00 ± 0.19	1.64 ± 0.30	1.13 ± 0.19	0.86 ± 0.11	0.09	0.08
*IFNLR1*	1.00 ± 0.08	0.82 ± 0.06	1.10 ± 0.16	0.93 ± 0.12	0.09	0.36
*TRIF*	1.00 ± 0.12	1.20 ± 0.17^a^	0.74 ± 0.17 ^b^	0.60 ± 0.08 ^b^	0.37	0.02
*TRAF3*	1.00 ± 0.19	0.96 ± 0.13	0.91 ± 0.18	0.76 ± 0.08	0.86	0.57

* Means different from CON (*p* < 0.05); ^a,b^ Means not sharing the same superscript differ at *p* < 0.05. The *p*^1^ means *t*-test between CON and CON-PEDV, and *p*^2^ value means multiple comparisons among the PEDV challenge groups.

## Supplementary Materials

The following are available online at https://www.mdpi.com/2076-2615/9/9/627/s1. Figure S1. Detection of jejunal mucosa porcine epidemic diarrhea virus (PEDV) infection by agarose gel electrophoresis. Treatment 1 means control without PEDV challenge. Treatment 2, 3, 4, 5, 6 means PEDV challenge with 5.5, 43.0, 80.5, 118.0, 155.5 μg 25(OH)D3/kg supplementation, respectively. PEDV primer: F: TTTCTAAGGTACTTGCAAATAATG; R: TTGGAGATCTGGACCTGTTGTTGC.
