# Supplementary material for: Dietary 25-Hydroxyvitamin D3 Supplementation Alleviates Porcine Epidemic Diarrhea Virus Infection by Improving Intestinal Structure and Immune Response in Weaned Pigs
Source: Animals (Basel). 2019 Aug 29;9(9):627. doi: 10.3390/ani9090627 (PMC6770734; doi:10.3390/ani9090627)
Supplement: Supplementary file 1 [file animals-09-00627-s001.pdf]

Supplementary Materials: Dietary 25-Hydroxyvitamin D3 Supplementation Alleviates Porcine Epidemic Diarrhea Virus Infection by Improving Intestinal Structure and Immune Response in Weaned Pigs

Jiwen Yang, Gang Tian, Daiwen Chen, Ping Zheng, Jie Yu, Xiangbing Mao, Jun He, Yuheng Luo, Junqiu Luo, Zhiqing Huang, Aimin Wu and Bing Yu *

Institute of Animal Nutrition, Sichuan Agricultural University, NO. 46 Xinkang Road, Yucheng District, Yaan, Sichuan 625014, China

* Correspondence: ybingtian@163.com; Tel.: +86-0835-288-5106

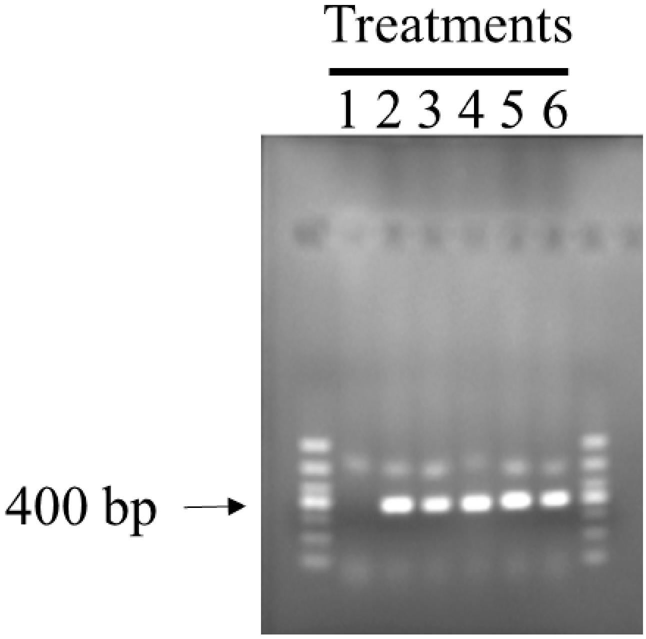

Figure S1. Detection of jejunal mucosa porcine epidemic diarrhea virus (PEDV) infection by agarose gel electrophoresis. Treatment 1 means control without PEDV challenge. Treatment 2, 3, 4, 5, 6 means PEDV challenge with 5.5, 43.0, 80.5, 118.0, 155.5 μg 25(OH)D_3/kg supplementation, respectively. PEDV primer: F: TTTCTAAGGTACTTGCAAATAATG; R: TTGGAGATCTGGACCTGTTGTTGC.

© 2019 by the authors. Licensee MDPI, Basel, Switzerland. This article is an open access article distributed under the terms and conditions of the Creative Commons Attribution (CC BY) license (http://creativecommons.org/licenses/by/4.0/).
